# Organochlorine Pesticides in Honey and Pollen Samples from Managed Colonies of the Honey Bee *Apis mellifera* Linnaeus and the Stingless Bee *Scaptotrigona mexicana* Guérin from Southern, Mexico

**DOI:** 10.3390/insects9020054

**Published:** 2018-05-10

**Authors:** Jovani Ruiz-Toledo, Rémy Vandame, Ricardo Alberto Castro-Chan, Rosa Patricia Penilla-Navarro, Jaime Gómez, Daniel Sánchez

**Affiliations:** 1 El Colegio de la Frontera Sur Unidad Tapachula, Carretera Antiguo Aeropuerto Km 2.5, Tapachula 30700, Chiapas, Mexico; jonrut.88b@gmail.com (J.R.-T.); rcastro@ecosur.mx (R.A.C.-C.); jgomez@ecosur.mx (J.G.); 2 El Colegio de la Frontera Sur Unidad San Cristóbal de las Casas, Periférico Sur s/n, María Auxiliadora, San Cristóbal de Las Casas 29290, Chiapas, Mexico; remy@ecosur.mx; 3 Centro Regional de Investigación en Salud Pública, Instituto Nacional de Salud Pública, Laboratorio de Resistencia a Insecticidas, 4a. Norte y 19 Calle Poniente S/N, Tapachula 30700, Chiapas, Mexico; penilla@insp.mx

**Keywords:** pesticides, native bee, persistency, biomonitoring

## Abstract

In this paper, we show the results of investigating the presence of organochlorine pesticides in honey and pollen samples from managed colonies of the honey bee, *Apis mellifera* L. and of the stingless bee *Scaptotrigona mexicana* Guérin. Three colonies of each species were moved into each of two sites. Three samples of pollen and three samples of honey were collected from each colony: the first collection occurred at the beginning of the study and the following ones at every six months during a year. Thus the total number of samples collected was 36 for honey (18 for *A. mellifera* and 18 for *S. mexicana*) and 36 for pollen (18 for *A. mellifera* and 18 for *S. mexicana*). We found that 88.44% and 93.33% of honey samples, and 22.22% and 100% of pollen samples of *S. mexicana* and *A. mellifera*, respectively, resulted positive to at least one organochlorine. The most abundant pesticides were Heptaclor (44% of the samples), γ-HCH (36%), DDT (19%), Endrin (18%) and DDE (11%). Despite the short foraging range of *S. mexicana*, the number of pesticides quantified in the honey samples was similar to that of *A. mellifera*. Paradoxically we found a small number of organochlorines in pollen samples of *S. mexicana* in comparison to *A. mellifera*, perhaps indicating a low abundance of pollen sources within the foraging range of this species.

## 1. Introduction

Bees are essential organisms that contribute enormously in preserving ecosystems and human well-being by both pollinating wild plants and increasing the productivity of crops [1]. Highly social species, like honey bees and stingless bees, also produce highly appreciated substances with well-deserved high value in the market, wax, propolis and mainly honey, which represent an important economic input for many countries [2]. Many social bees manage to store several kilograms of such products by means of food recruitment communication: they fly several kilometers (up to 5 km in the case of larger species) from the colony, return safely and inform nestmates of the location of sources of nectar, pollen and resins. This way, the foraging force of a colony focuses in certain patches, instead of wandering around looking for food [3,4,5,6]. Unfortunately, since it is not uncommon for bees to share space with agroecosystems, food recruitment communication might increase the likelihood of transporting agrochemicals like pesticides to the colony [7,8,9], as demonstrated in *A. mellifera* [10], causing several deleterious effects in bee populations, either chronic or acute [11,12,13,14].

Control of pests of agricultural importance is currently thought to be better approached by an integrated pest management scheme, in which cultural, biological and chemical strategies, if used judiciously, are supposed to reduce pesticide usage and thus the threat to non-target organisms like bees [15]. However, the most common practices involve the use of chemicals alone, which might pose serious negative effects upon human and environmental health. Acute poisoning of beneficial arthropods during application of pesticides is of current concern, but long term exposure to minute, sublethal, amounts in resources collected by bees can cause a slow decline in the populations of these species. Sublethal exposure to pesticides is known to affect several essential physiological and behavioral functions, like reproduction and learning [16,17,18,19], homing flight [20,21,22,23,24] and locomotion [25,26,27], which are key functions for foraging. This has taken research to develop new molecules that degrade quickly in the environment and are more effective against specific pests. Examples of this are found among organophosphorus pesticides, pyrethroids and macrolides [28,29]. However, they are more expensive that older alternatives, like organochlorines which, despite the ban in many parts of the world, are still under heavy production and use in developing countries, or in the best of the cases, they were just recently forbidden, like in Mexico [30].

Organochlorine pesticides (OCs) are characterized by their high chemical stability, low water solubility, high solubility in organic solvents and characteristic resistance to chemical and biological degradation [31]. Such features allow OCs to bioaccumulate and biomagnificate in animal fatty bodies [32,33,34]. Among the most persistent OCs we can find: DDT (half-lifetime: 10 to 15 years), toxaphene (12 years), endrin (10 years), chlordane (8 years), dieldrin (7 years), aldrin (5 years), heptachlor (4 years) and lindane (2 years) [32,35]. In Mexico, the use of OCs began in the 1940s with the arrival of the Green Revolution [36,37], and played key roles both in agriculture and in public health programs. However, research showed both several human health disorders and a reduction in fauna associated with OCs exposure, so banning began as early as in the 1970s [31,38], but their effects are still present [31,38].

The state of Chiapas, in southern Mexico, was one of the last states in which DDT was banned both in agriculture and in public health [39,40]. In the Soconusco region, Chiapas, OCs were used for more than 40 years for pest control in coffee, but mostly in cotton crops (Table 1). The area devoted to cotton cultivation grew remarkably, from 518 to 35,227 ha cultivated in 1950–1978 [41], after the implementation of modern agricultural technologies, particularly the use of insecticides. Official records indicate that a mixture of toxaphene and DDT was sprayed in doses of 6–7 L/ha per cycle [42]. Catalán [41] reported that the application of toxaphene/DDT was as frequent as 21 times per crop cycle; moreover, when the total area under cotton cultivation was the largest, the amount of OCs that was applied peaked 1,109,650.5 L/year. Regarding public health, the Soconusco region is also known for the high incidence of malaria, so DDT was also used to control vector-borne diseases, with up to one monthly application of 2 g/m^2^ of active ingredient on average [43,44]. It is estimated that 69,545 ton of DDT were used just during the Mexican health campaigns in 1957–2000 [45].

It is also important to recall that crop phenology, the life cycle of insect pests, and local climatic conditions might reduce the efficacy of pesticides along time and space. Thus it is not surprising that several studies had reported seasonal and spatial variation in pesticide availability in many types of matrixes. For example, Helm et al. [46] showed a seasonal trend of polychlorinated naphthalene’s in Canada, Russia and USA, with highest levels occurring during Winter; Eqani et al. [47] also found higher concentrations of OCs and polychlorinated biphenyls in fishes during the winter season in Pakistan and China. These results suggest that bees are also exposed to variable concentrations of pesticides, which, along with the availability of food sources, can have variable impacts in the survival of these species over time. Moreover, biological differences among bee species may be very important when evaluating their chances of survival under identical environmental conditions, v.gr. larger species can fly longer distances to forage and thus the probability of finding food sources contaminated with pesticides is higher, while in smaller species the opposite situation might occur.

The aim of this study was to investigate the seasonal and the spatial variation in the levels of organochlorine pesticides present, if any, in honey and pollen of managed colonies of both the honey bee *Apis mellifera* L. and the stingless bee *Scaptotrigona mexicana* Guérin in a highly fragmented landscape. We hypothesize that honey and pollen samples from *A. mellifera* colonies will have more pesticides (and in higher amounts) than samples from *S. mexicana* colonies because *A. mellifera* is a larger species with a greater foraging range [57,58], features that increase the exposure to contaminated resources. Also we expect that colonies located in sites with a larger proportion of land devoted to intensive agricultural activities will have more OCs (and in higher amounts) than in sites with a smaller proportion of land devoted to agriculture.

## 2. Materials and Methods

### 2.1. Study Area

The study was carried out in the city of Tapachula, located in the Soconusco region, Chiapas in southern Mexico, between June 2015 and May 2016. We chose two sites according to the proportion of land used for agriculture: site 1 is characterized by 2% of urban settlements, 36% of conserved remnants of original forest and 62% of agriculture (soybean and mango); site 2 has 1% of land occupied by urban settlements, 17% covered with original forest and 82% used to grow soybeans and mango. Six managed colonies of each species were selected at random and moved to two locations, three colonies of each species in each location. Pollen and honey from each colony was collected three times: June 2015, November 2015 and May 2016. All colonies used in this study were considered healthy after a careful, qualitative inspection by two certified beekeepers, one specialized on stingless bees and the other on honey bees, who evaluated colony strength, foraging force, brood amount, defensiveness and food (pollen and honey) reserves.

### 2.2. Sample Collection

Pollen samples from honey bee colonies were collected by using pollen traps placed at the entrance of the hives. Next, samples were placed in 15 mL Falcon tubes for transportation [59]; honey was squeezed from the comb into a 50 mL polyethylene Falcon tube. Honey and pollen samples from *S. mexicana* were obtained from pots inside the colony using a sterilized syringe or a spoon, respectively, and transported in 15-mL Falcon tubes [60]. All samples were placed inside a cool box with ice packs, taken to the laboratory and frozen at −20 °C until extraction. The total number of honey samples collected was 36 (18 for *A. mellifera* and 18 for *S. mexicana*) and 36 of pollen (18 for *A. mellifera* and 18 for *S. mexicana*).

### 2.3. Organochlorine Extraction

Organochlorine residues were extracted according to the methodology by Wiest et al. [61]. Five grams of honey or two grams of pollen were weighed in a 50 mL centrifuge tube, 10 mL of water were added and the mixture was vigorously shaken to dissolve honey. Next, 10 mL of acetonitrile (and 3 mL of hexane in the case of pollen), 4 g of anhydrous MgSO_4_, 1 g of sodium chloride, 1 g of sodium citrate dihydrate and 500 mg of disodium citrate sesquihydrate were added; the tube was immediately shaken by hand, vortexed for one minute, and then centrifuged for 2 min at 5000× *g*. Six mL of supernatant were transferred into a 15 mL PSA (primary secondary amine) tube that contained 900 mg of anhydrous MgSO_4_, 150 mg of PSA bonded silica and 150 mg of C18 bonded silica. This tube was immediately shaken by hand, vortexed for 10 s and centrifuged for 2 min at 5000× *g*. Finally, 4 mL of the extract were transferred into a 10 mL glass, cone-ended centrifuge tube, evaporated until a final volume of 50 μL and kept at −18 °C until analysis. Agilent Technologies, Santa Clara, CA, USA, provided all salts used for OCs extraction.

### 2.4. Chemicals

For validation and standardization of the analytical method we used a mixture of standard-grade OCs from Sigma-Aldrich (Reference 4S7426-U: St. Louis, MO, USA): aldrin, β-HCH, α-HCH, γ-HCH, δ-HCH, p,p′-DDD, p,p′-DDE, p,p′-DDT, Dieldrin, α-endosulfan, β-endosulfan, Endosulfan sulfate, Endrin, Endrin aldehyde, Heptachlor, and Heptachlor epoxide. Organic solvents used for extraction were HPLC-grade and were supplied by Sigma-Aldrich.

### 2.5. Gas Chromatography Analysis

The extracts were analyzed by gas chromatography using a Perkin Elmer Clarus 500 (Shelton, CT, USA) gas chromatograph, equipped with an electron capture detector, autosampler, and a programmable split/splitless injector. The injection volume of extract was 2 μL in splitless mode. The initial temperature of the injector was set at 120 °C, and the speed of the carrier gas (hydrogen) was fixed at 48 cm/s. The temperature of the detector was 350 °C, and the make-up flow was 30 mL/min. An Agilent J & W DB-35MS column (p/n 122–3832) of 30 m length, 0.250 mm inner diameter, and 0.25 μm film thickness was used. The initial oven temperature was 110 °C, which was maintained for 1.4 min, followed by a temperature ramp with increments of 13 °C/min up to 285 °C, holding at 285 °C for 1 min, another ramp of 30 °C/min up to 300 °C, and holding until the end of the routine (3 min). The total time of the analysis was 19.36 min.

Under previous described conditions the residue levels of OCs were quantitatively determined by the external standard method using peak area. The limits of detection (LOD) and quantification (LOQ) were determined by the least squares regression method on the OC curves using data generated by nine replicates near the lowest concentration attainable on the calibration curve. The curves of all the standards (analytes) were situated within the acceptable limits of the linearity criterion (r = 0.99). The LOD values of the different OCs in honey fell between 0.175–1.174 ng/g; for pollen the LOD values fell between 0.008–0.058 ng/g. The LOQ values of the different OCs in honey fell between 0.585–3.913 ng/g; for pollen the LOQ values fell between 0.029–0.195 ng/g. Recoveries were determined by spiking with the surrogate standards prior to extraction. Amounts were similar to detected quantities of analytes in the samples. The percentage of recovery was determined by adding the levels of the analyte kit to the honey and pollen samples (in triplicate); the range of the average percentage of recovery in pollen was 78.3 to 103% and in honey from 79.1 to 106.8%.

### 2.6. Hazard Characterization and Statistical Analysis

A descriptive analysis of the levels of OCs was conducted by calculating geometric means, median, standard deviations and minimum and maximum. In order to find any statistical difference among sampling dates and between sites we carried out a general linear mixed model ANOVA, in which colony was considered a random effect and site and date of sampling as fixed effects. All statistics were calculated using the R software package at a significance level of 0.05 [62].

We calculated the hazard quotient (HQ) for honey bees for each single pesticide using the methods described by Stoner and Eitzer [63] and Traynor et al. [64]. Briefly, the HQ was calculated by dividing the average amount (μg/kg) of each pesticide by its respective oral LD50 (μg/bee) available from the US EPA Ecotox Database (http://cfpub.epa.gov/ecotox/) and the University of Hertfordshire Pesticide Properties DataBase (PPDB, http://sitem.herts.ac.uk/aeru/ppdb/en/index.htm). We estimated the HQ of pollen and honey samples, which typically contained multiple residues, assuming additive toxic effects. We excluded possible synergistic or antagonistic effects due to lack of quantitative data on interactions between most of the pesticides under assessment [8,64]. Based upon the average daily pollen consumption of a nurse bee (9.5 mg/bee/day) [65,66] and the total nectar consumption rate (TFR) for adult worker bees (292 mg/bee/day) [67], a HQ of 1000 corresponds to consuming 1% of the median lethal dose (LD50) per day. For *S. mexicana* it was not possible to calculate the HQ due to the absence of data regarding the LD50 of OCs in this species.

Maximum amount of residues detected in pollen and honey samples were compared with the acute reference dose (ARfD, the amount of a chemical that can be consumed by a person at one meal or on one day that would lead to no harm), the acceptable daily intake (ADI, is the quantity of a chemical that can be consumed every day for a life-time causing no harm), and the maximum residue limit (MRL, the maximum concentration of a pesticide legally permitted in or on food commodities or animal feeds) [68,69,70].

## 3. Results

Overall, we detected 15 out of the 16 organochlorine compounds included in our screening list (Table 2 and Table 3). We found that 22.22% of *S. mexicana* pollen, 88.33% of *S. mexicana* honey, 100% of *A. mellifera* pollen and 94.44% of *A. mellifera* honey samples were positive to at least one organochlorine. Thus there was variation in the amount and identity of the pesticides detected in the different matrixes, colonies, species and dates. Heptachlor was the most frequent OC (found in 88% of all samples) followed by Endrin (66%), γ-HCH (61%), DDT (50%) and Heptachlor epoxide (44%) (Table 2 and Table 3).

In the case of honey samples, 14 OCs were detected in *A. mellifera* and 9 in *S. mexicana* samples. The highest concentrations (mean ± SD μg/kg) corresponded to Endrin (10,032.14 ± 1485.93), Heptachlor (794.83 ± 484.81) and p,p′-DDD (78.73 ± 9.27). The honey samples obtained from colony 1 of *A. mellifera* had the highest concentrations of pesticides during the study period, followed by colony 4 and colony 5. Regarding pollen samples, we detected 9 and 3 OCs in *A. mellifera* and *S. mexicana* samples, respectively. p,p′-DDE (2696.98 ± 420.32), Heptachlor (2570.31 ± 484.81 μ/kg) and Heptachlor epoxide (699.26 ± 104.54 μg/kg) had the highest concentrations. For *S. mexicana* colonies six (645.08 μg/kg), five (440.77 μg/kg) and four (152.36 μg/kg) had the highest concentrations, and for *A. mellifera* colonies three (10,032.14 μg/kg), four (3996.87 μg/kg) and five (2246.54 μg/kg) had the highest amounts (Table 2 and Table 3).

For *S. mexicana*, 100% of the honey samples collected on all dates showed at least one organochlorine. In the case of A. mellifera, only 33% of the samples were positive. The pollen samples in both species showed a higher percentage of samples with at least one organochlorine during the collection dates (Table 4).

For *S. mexicana*, the most abundant pesticide was γ-HCH: four pollen and nine honey samples contained it (22.22% and 50% of all samples, respectively); in the case of *A. mellifera*, thirteen pollen samples were positive to γ-HCH (72.22%). Heptachlor was detected in fifteen honey samples (83.33%) (Table 2 and Table 3). All *S. mexicana* and *A. mellifera* colonies survived, and no visible sign of weakening could be observed at the end of the study.

For statistical comparison we analyzed only those OCs that (a) were detected in all sampling dates; (b) were detected in both sites; (c) had at least nine positive samples and (d) had at least two positive samples in each date or site. The OCs that fitted these criterions, among dates, were Heptachlor, p,p′-DDT, Endrin and γ-HCH. Heptachlor, γ-HCH, α-Endosulfan, p,p′-DDE and Endrin fit these criterions in both sites.

We found significant differences in the concentrations of, p,p′-DDT (χ^2^ = 10.924, df = 2, *p* < 0.004), Endrin (χ^2^ = 6.782, df = 2, *p* < 0.033) and γ-HCH (χ^2^ = 18.5, df = 2, *p* < 0.001) among dates. Heptachlor concentrations showed no significant difference (Table 4). Regarding sites, only α-Endosulfan (χ^2^ = 4.59, df = 1, *p* = 0.032) and p,p′-DDE (χ^2^ = 6.794, df = 1, *p* = 0.009) showed significant differences. The concentrations of γ-HCH, heptachlor and Endrin did not show significant difference.

For *A. mellifera* the HQ was higher than 500 in 5% of the positive pollen samples, and 47% had an HQ higher than 1000. Five percent of the honey samples had HQ superior to 500, while 28% were higher than 1000. In the case of *S. mexicana*, 13% and 6% of the honey samples had HQ values higher than 500 and 1000, respectively. Endrin (HQ = 21,806) and Heptachlor (HQ = 1511) had the highest HQ values in *A. mellifera* pollen samples. Heptachlor (HQ = 4886) and Dieldrin (HQ = 339) had the highest values in the honey samples (Table 2 and Table 3).

The organochlorine residues reported here exceeded the EU safety and legal levels of pesticide in pollen and honey (ARfD, ADI and MRL) (Table 5). All the organochlorine residues from the honey and pollen samples of both bee species were superior to the ADI. The DDT residues exceeded all reference levels. A high proportion of samples with γ-HCH and α-endosulfan exceeded the acceptable limits (Table 5).

## 4. Discussion

In this study, we aimed to quantify organochlorine pesticides in honey and pollen samples from two bee species, the honey bee *A. mellifera* and the small-sized stingless bee *S. mexicana*, which have putatively different flight ranges. All samples had at least one organochlorine, but the pollen samples from honey bees had the higher number of pesticides. Slight differences in the number and identity of some pesticides between sampling sites and dates were found, but no pattern could be discerned.

It is not uncommon to find honey bee products like honey, pollen and wax, from colonies located in agricultural landscapes, contaminated with pesticides [71,72,73] in countries like Argentina [74], Belgium [75], Brazil [76], China [77], Colombia [60], Egypt [78], France [61], Germany [79], Greece [80], India [81], Italy [8], Mexico [82], Poland [83], Portugal and Spain [84], Turkey [85] and USA [73]. However, given the seasonal variation in the agricultural activities related to insecticide application, climatic factors, among others, not all samples show evidence of contact with such chemicals; for instance, Balayiannis and Balayiannis [86] found organophosphorous insecticides in 56% of their honey samples; Mullin et al. [73], Pohorecka et al. [87] and Panseri [88] reported that 60%, 50% and 94% of their samples (wax, pollen, honey and bee workers) had residues of at least two pesticides. We found no clear correlation between date, site and colony and the presence of pesticides; actually we failed to carry out a survey about the strategy that local growers adopt to apply insecticides to control pest, i.e., whether they constantly monitor pest abundance or they carry out preventive measures and apply insecticides regardless pest abundance. Additionally, it is important to consider the location of our colonies in the agricultural landscape, since its closeness to farms determines the likelihood that bee workers find pesticides [89]; this has yet to be investigated.

Spatial and temporal variations in the identity and amount of pesticides have been documented. Chauzat et al. [90] reported that in 2002–2005 in France, there was variation in the concentrations of several pesticides; Pirard et al. [75], Nguyen et al. [91], Mullin et al. [73], García-Chao et al. [92], Bernal et al. [93] and Valdovinos-Flores et al. [82], also found, in different countries, variations in the amount and identity of pesticides in distant localities. The most common chemicals include fungicides, herbicides [73,93,94,95], neonicotinoids [59,90,94,96], organophosphorous [78], organochlorines and pyretroids [30]. Given the reduced use of neonicotinoids in our study area and the short halftime of organophosphorous insecticides we focused on organochlorines.

The concentration of the pesticides in our study also varied between sites, date of sampling, colonies and type of sample. Many studies have also shown a similar pattern. For instance, Mullin et al. [73] reported 121 pesticides (and metabolites) at concentrations ranging 99–204 ppm in pollen, wax and worker samples; Santos de Azebedo [97] found aldrin (2 ng/g), endosulfan (1–33 ng/g), endrin aldehyde, heptachlor epoxide, p,p′-DDE and p,p′-DDT (1–94 ng/g) in pollen samples; Panseri et al. [88] found DDT, DDD and DDE in honey samples at 8.8 ng/g, 1.9 ng/g and 15.4 ± 0.3 ng/g, respectively; Malhat et al. [30] documented that a honey sample had γ-HCH at a concentration higher than the ARfD, ADI and MRL (Table 5); in Mexico, Valdovinos-Flores et al. [82] reported samples of honey and wax contaminated with 93 pesticides, including DDT at 0.175 mg/kg. In our study, we found endrin (10 mg/kg), p,p′-DDE (2.7 mg/kg) and heptachlor (2.6 mg/kg) to be the most abundant pesticides, even at concentrations higher than reported in other works. Surprisingly the concentrations we found are close to the LD50 calculated for honeybees, but our colonies did not show any clear sign of depopulation or reduced activity [98].

It has been shown that the amount and variety of pesticides in honey bee colonies is closely related to the closeness to the source of contamination and the length of the exposure [30,73,88,90,92,94]. Other important factor is the half-life of the pesticides; organochlorine pesticides have been banned for decades in many countries, including Mexico, but residues are still present. Table 2 and Table 3 show that DDT, DDE and DDD can be found in the study zone. It is possible to estimate how recent are DDT applications by calculating the DDE/DDT ratio [99]. It is worrying to discover that our data reveals that it is highly likely that DDT is still used despite the ban (DDE/DDT ratio = 0.0027–1.049), so the current policies are not efficient in controlling the production and marketing of organochlorines in this part of Mexico.

Pollen samples from *A. mellifera* had the highest number of pesticides (14 out of 16) with the highest concentrations, so they represent a closer view of the situation regarding OCs in the study area. Chauzat et al. [59] also pointed out that pollen is the best matrix to determine the presence of pesticides. Moreover, other studies have indicated that the high octanol/water partition coefficient (log K_ow_) of pesticides allows them to be absorbed by pollen more easily than by honey [89]. *Scaptotrigona mexicana* pollen and honey samples only resulted positive to 4 and 9 OCs, respectively. Such difference might be the result of the shorter foraging range of *S. mexicana*; it is estimated that 10000–25,000 honey bee workers make 10 round trips to collect food, and that the exploring area covers up to 10 km^2^ around the colony [76,100,101,102]. Thus, the likelihood that foragers encounter pesticides is high [103]. In the case of *S. mexicana* it is known that, given its size and the smaller number of workers compared to *A. mellifera*, the foraging range is far shorter than for *A. mellifera* [104], thus reducing the exposure to a lesser degree.

Organochlorine pesticides persist in the environment, and tend to bioaccumulate in plants from polluted soil. OCs can enter the food chain mainly via fatty products, but they can also be distributed via non-fatty products such as honey, reaching higher concentrations at high trophic levels, causing undesirable impacts and anomalies in food webs [88,105]. For example, DDE levels cause hyporeflexia in infants [106] and egg-shell thinning in some birds [107], while Aldrin and Dieldrin are carcinogens, and Lindane is correlated with the formation of liver tumors in mice [108]. In our study, some OCs had concentrations that exceeded the legally acceptable limits; almost 100% of samples with γ-HCH and Heptachlor had levels above ARfD, ADI and MRL. This is worrisome since many of these pesticides are still used despite their prohibition. In Mexico, cancer and leukemia are the second cause of mortality in children aged 2 to 15 years-old [109], and incidence is rising. There is some circumstantial evidence that OCs could be involved in this issue.

We found three to fourteen OCs in a single colony, meaning that potential additive, or even worse, synergistic negative effects can be at work [110]. Actually, current agricultural practices facilitate the exposure of bees to mixtures of pesticides by multiple routes [103,111,112,113,114]. In our study region, different pesticides are employed against common pests and applied in different amounts all year around; and it seems to be the case worldwide [8,30]. For this reason, the EPA has recommended a modeling approach to add all exposure risks for bees, with special emphasis in the differential sensitivity of the life stage and probability of exposure [115]. For example, a synergistic interaction between pyrethroid insecticides and the demethylase inhibitor (DMI) fungicides (e.g., Triflumizole) has been demonstrated in honey bees. Triflumizole increases the toxicity of the insecticide by delaying metabolism and detoxification processes [116,117]. Some neonicotinoids are thought to interact similarly with this group of fungicides. When applied in a laboratory setting, DMI fungicides increased the toxicity of Acetamiprid and Thiacloprid as much as 244-fold, but not Imidacloprid [118]. However, when honey bees were exposed to foliage treated with Acetamiprid and Triflumizole under semifield conditions, no differences in mortality rates were seen [118]. Similarly, Schmuck et al. [119] found that DMI fungicides increased toxicity of Thiacloprid to honey bees significantly in the laboratory, but no adverse effects were seen in bees exposed to sprayed vegetation in a semifield setting. The exposure of bees to these mixtures in different concentrations could generate a greater risk, especially to persistent pesticides [73,120]. That is why it is necessary to understand how to position conservation efforts in the landscape with healthy populations of bees to guide the best management practices and optimize the health of pollinators by improving sustainability in agroecosystems.

The use of organochlorine pesticides has been curtailed in Mexico since early 1970s. Considering the long span of time since this ban was enforced, it might be difficult to assume that residues of organochlorine pesticides have been persisting intact without any metabolic change for more than 40 years. Therefore it is safe to assume that residues detected in this study are the result of recent applications. A number of organochlorine pesticides are continuously used illegally in the Soconusco region, so the concentrations of these pesticides persist in the environment and might increase, meaning a greater risk for bees. This situation is not limited to developing countries like Mexico. Chauzat et al. [59] recently reported two banned pesticides in France, Coumaphos and Lindane. Likewise, Tosi et al. [8] showed that banned pesticides are still in use; these authors found an organophosphate insecticide banned in Italy since 2003, a carbamate insecticide banned since 2007 and an organophosphate insecticide banned since 2006 in the EU. In addition, Malhat et al. [30] reported the presence of banned organochlorine pesticides since 1970, so they reasoned that the pesticide residues in the study area were the result of recent applications.

## 5. Conclusions

Our data provide a clear indication of the widespread use of OCs in the study area, confirming honey bee and beehive matrixes as appropriate sentinels for bioenvironmental monitoring. This could be an effective tool for beekeepers to select production areas in particular for the production of organic honey. In addition, this study revealed, for the first time, that bees in our study area are exposed to OCs pesticides, which represents a risk to both bees and human health by consumption of bee products such as honey [7,121,122]. Our study has unexpectedly revealed the presence of a wide spectrum of OC compounds, even though it is not officially used in Chiapas from the 2000’s. Efforts should be directed to find out the source of these compounds and to make sure none of them is being smuggled into the country. However, more studies conducted in other parts of Mexico might be necessary in order to determine the actual the prevalence of these pesticides residues, and their impact on the honey industry and the safety of consumers.

## Figures and Tables

**Table 1:** Organochlorine residues reported in the Soconusco region, Chiapas, México.

Sample	Organochlorine Pesticide	Concentration	Reference
Water	DDD *	2 μg/L	Hernández-Romero et al. [48]
Sediments (lagoon system)	DDE **	247 ng/L	Hernández-Romero et al. [48]
	Endosulfan	814 ng/L	
Air	Chlordane	5.8–12 pg/m^3^	Alegría et al. [40]
	Toxaphene	6.2–229 pg/m^3^	
	Dieldrin	0.9–11 pg/m^3^	
	Endosulfan	92–341 pg/m^3^	
	DDT	239–2360 pg/m^3^	
Soil	Chlordane	<0.0033–2.7 ng/g	Wong et al. [49]
	Toxaphene	<LD—334 ng/g	
	Endosulfan	<LD—909 ng/g	
	DDT	<LD—360 ng/g	
Fish	DDT	373.67–1937.90 ng/g lipids	Herrera-Portugal et al. [50]
Cheese	DDT	7.0–10.87 ng/g lipids	Herrera-Portugal et al. [51]
	DDD	1.11–3.71 ng/g lipids	
	DDE	17.0–38.52 ng/g lipids	
Blood plasma	DDT	67.4 μg/L	Barraza-Villareal et al. [52]
		nd—46.76 μg/L	Herrera-Portugal et al. [43]
		12.08 ± 8.58 μg/L	Herrera-Portugal et al. [53]
		50.2 ng/mL	Pérez-Maldonado et al. [44]
		15.4–17,886.5 ng/g lipid	Trejo-Acevedo et al. [54]
		1596.4 ng/g Lipid	Rivero-Pérez et al. [55]
		6.37–29.66 μg/L	Ruiz-Suárez et al. [56]
	DDE	nd—68.09 μg/L	Herrera-Portugal et al. [43]
		53.32 ± 35.61 μg/L	Herrera-Portugal et al. [53]
		203.5 μg/L	Barraza-Villareal et al. [52]
		3213.8 ng/g Lipid	Trejo-Acevedo et al. [54]
		15,457 ng/g Lipid	Rivero-Pérez et al. [55]
		1.1–222.6 μg/L	Ruiz-Suárez et al. [56]
	γ-HCH	351.1–6153.8 ng/g lipid	Trejo-Acevedo et al. [54]
		1596.4 ng/g Lipid	Rivero-Pérez et al. [55]
	γ-HCH	0.77–6.25 μg/L	Ruiz-Suárez et al. [56]
	β-HCH	2.03–8.74 μg/L	
	Heptachlor	1.74–4.40 μg/L	
	β-endosulfan	0.70–43.90 μg/L	
	Endrin aldehyde	0.51–6.76 μg/L	

* DDD (1,1-dichloro-2,2-bis(p-chlorophenyl)ethane), ** DDE (1,1-dichloro-2,2-bis(p-chlorophenyl)ethylene).

**Table 2:** Concentration of OCs in honey samples of *A. mellifera* (Amell) and *S. mexicana* (Smex).

Analyte	N	% ≥ DL ^a^		GM ^b^		Median		SD		Minimum		Maximum		HQ	
		*Amell*	*Smex*	*Amell*	*Smex*	*Amell*	*Smex*	*Amell*	*Smex*	*Amell*	*Smex*	*Amell*	*Smex*	*Amell*	*Smex*
α-HCH	18	11.11	11.11	0.51	0.48	6.54	4.28	0.51	0.33	5.46	3.8	7.63	4.79	N/C	N/C
γ-HCH	18	61.11	50	9.95	37.36	30.18	52.22	9.95	14.29	5.43	8.8	143.14	207.15	35.56	N/C
β-HCH	18	16.67	22.22	3.83	9.55	43.87	38.69	3.83	4.71	22.57	26.1	53.3	68.41	nc	N/C
Heptachlor	18	83.33	44.44	199.54	106.53	117.79	131.73	199.54	45.03	24.35	96.4	2570.32	645.08	178.71	N/C
δ-HCH	18	0	0	N/D	N/D	N/D	N/D	N/D	N/D	N/D	N/D	N/D	N/D	-	-
Aldrin	18	0	0	N/D	N/D	N/D	N/D	N/D	N/D	N/D	N/D	N/D	N/D	-	-
Heptachlor epoxide	18	38.89	16.67	80.401	3.37	52.06	20.83	47.27	1.83	13.89	18.1	699.26	21.68	N/C	N/C
α-Endosulfan	18	33.33	11.11	16.27	6.12	18.01	55.05	11.32	4.21	4.77	51	204.27	59.12	21.88	N/C
p,p′-DDE	18	22.22	11.11	370.56	3.29	1543.58	29.6	187.05	2.29	885.98	25.1	2696.98	34.1	N/C	N/C
Dieldrin	18	11.11	0	3.488	N/D	31.4	N/D	2.71	N/D	15.72	N/D	47.06	N/D	27.21	N/D
Endrin	18	0	0	N/D	N/D	N/D	N/D	N/D	N/D	N/D	N/D	N/D	N/D	-	-
p,p′-DDD	18	0	0	N/D	N/D	N/D	N/D	N/D	N/D	N/D	N/D	N/D	N/D	-	-
β-Endosulfan	18	11.11	0	1.12	N/D	10.13	N/D	0.85	N/D	5.78	N/D	14.4753	N/D	9.15	-
p,p-DDT	18	0	16.67	N/D	44.09	N/D	253.82	N/D	27.62	N/D	99	N/D	440.78	-	N/C
Endrin aldehyde	18	0	16.67	N/D	6.49	N/D	35.64	N/D	3.58	N/D	33.2	N/D	47.96	-	N/C
Endosulfan sulfate	18	0	0	N/D	N/D	N/D	N/D	N/D	N/D	N/D	N/D	N/D	N/D	-	N/D

Concentration in honey reported in μg/Kg; ^a^ % of samples with detectable levels (% ≥ DL); ^b^ values reported as geometric mean (GM); (SD) standard deviation; (N/D) not detected. (HQ) Hazard quotient; (N/C) not calculated.

**Table 3:** Concentration of OCs in pollen samples of *A. mellifera* (Amell) and *S. mexicana* (Smex).

Analyte	N	% ≥ DL ^a^		GM ^b^		Median		SD		Minimun		Maximun		HQ	
		*Amell*	*Smex*	*Amell*	*Smex*	*Amell*	*Smex*	*Amell*	*Smex*	*Amell*	*Smex*	*Amell*	*Smex*	*Amell*	*Smex*
α-HCH	18	16.67	0	2.9	nd	14.08	nd	1.93	nd	5.44	nd	32.61	N/D	N/C	-
γ-HCH	18	11.11	22.22	2.19	4.45	19.71	17.92	1.81	2.24	7.31	11.8	32.11	32.49	27.26	N/C
β-HCH	18	0	0	N/D	N/D	N/D	N/D	N/D	N/D	N/D	N/D	N/D	N/D	-	-
Heptachlor	18	38.89	11.11	140.65	1.75	415.48	15.72	60.95	1.45	35.9	5.7	794.83	25.77	386.53	N/C
δ-HCH	18	0	0	N/D	N/D	N/D	N/D	N/D	N/D	N/D	N/D	N/D	N/D	-	-
Aldrin	18	5.56	0	0.71	N/D	12.69	N/D	0.71	N/D	12.69	N/D	12.69	N/D	N/C	-
Heptachlor epoxide	18	44.44	0	17.35	N/D	19.48	N/D	7.08	N/D	10.33	N/D	92.22	N/D	N/C	-
α-Endosulfan	18	5.56	0	2.16	N/D	38.93	N/D	2.16	N/D	38.93	N/D	38.93	N/D	4.98	-
p,p′-DDE	18	11.11	0	2.32	N/D	20.85	N/D	1.79	N/D	10.61	N/D	31.09	N/D	N/C	-
Dieldrin	18	5.56	0	1.49	N/D	26.85	N/D	1.49	N/D	26.85	N/D	26.85	N/D	193.17	-
Endrin	18	72.22	0	1393.01	N/D	116.79	N/D	653.23	N/D	29.83	N/D	10,032.14	N/D	768.85	-
p,p′-DDD	18	5.56	0	4.37	N/D	78.73	N/D	4.37	N/D	78.73	N/D	78.73	N/D	N/C	-
β-Endosulfan	18	5.56	0	2.6	N/D	46.83	N/D	2.6	N/D	46.83	N/D	46.83	N/D	57.11	-
p,p-DDT	18	50	11.11	74.18	5.2	143.85	46.82	19.17	3.89	124.87	27.7	219.35	65.96	265.44	N/C
Endrin aldehyde	18	16.67	0	8.67	N/D	56.695	N/D	5.07	N/D	34.77	N/D	78.62	N/D	N/C	-
Endosulfan sulfate	18	5.56	0	2.47	N/D	44.45	N/D	2.47	N/D	44.45	N/D	44.45	N/D	N/C	-

Concentration in pollen reported in μg/Kg; ^a^ % of samples with detectable levels (% ≥ DL); ^b^ values reported as geometric mean (GM); (SD) standard deviation; (nd) not detected; (HQ) Hazard quotient; (nc) not calculated.

**Table 4:** Number of positive samples (% positive samples) in each date.

Date	Honey		Pollen	
	*S. mexicana*	*A. mellifera*	*S. mexicana*	*A. mellifera*
1 June 2015	6 (100%)	2 (33%)	2 (33%)	6 (100%)
1 November 2015	6 (100%)	2 (33%)	6 (100%)	4 (67%)
1 May 2016	6 (100%)	0 (0%)	6 (100%)	6 (100%)

**Table 5:** The total number of samples exceeding ARfD (acute reference dose), ADI (acceptable daily intake), and MRL (maximum residue limit) levels.

Analyte	ARfD	ADI	MRL	Pollen						Honey					
				Exceeding ARfD		Exceeding ADI		Exceeding MRL		Exceeding ARfD		Exceeding ADI		Exceeding MRL	
				*Amell*	*Smex*	*Amell*	*Smex*	*Amell*	*Smex*	*Amell*	*Smex*	*Amell*	*Smex*	*Amell*	*Smex*
α-HCH	N/R	N/R	0.01	-	-	-	-	100%	N/D	-	-	-	-	100%	100%
γ-HCH	60	5	8	0%	0%	100%	100%	50%	100%	54%	100%	100%	100%	91%	100%
Heptachlor	N/R	0.1	0.01	-	-	100%	100%	100%	50%	-	-	100%	100%	100%	100%
δ-HCH	N/R	N/R	0.01	N/D	N/D	N/D	N/D	N/D	N/D	N/D	N/D	N/D	N/D	N/D	N/D
Aldrin	N/R	0.1	0.01	-	-	100%	N/D	100%	N/D	-	-	-	-	N/D	N/D
Heptachlor epoxide	N/R	N/R	0.01	-	-	-	-	100%	N/D	-	-	-	-	100%	100%
α-Endosulfan	6	20	50	100%	N/D	100%	N/D	0%	N/D	84%	100%	N/D	100%	16%	100%
p,p′-DDE	N/R	N/R	0.05	-	-	-	-	0%	N/D	-	-	-	-	100%	100%
Dieldrin	N/R	0.1	0.01	-	-	100%	N/D	100%	N/D	-	-	100%	N/D	100%	N/D
Endrin	N/R	0.2	0.01	-	-	100%	N/D	100%	N/D	-	-	-	-	N/D	N/D
p,p′-DDD	N/R	N/R	0.05	-	-	-	-	100%	N/D	-	-	-	-	N/D	N/D
DDT	N/R	10	10	-	-	100%	100%	100%	100%	-	-	N/D	100%	N/D	100%

ARfD, ADI and MRL reported in μg/kg body weight. Exceeding values reported in % of samples that are above the reference values; (N/D) not detected; (N/R) not reported.
